# A novel sweet potato potyvirus open reading frame (ORF) is expressed via polymerase slippage and suppresses RNA silencing

**DOI:** 10.1111/mpp.12366

**Published:** 2016-04-28

**Authors:** Milton Untiveros, Allan Olspert, Katrin Artola, Andrew E. Firth, Jan F. Kreuze, Jari P. T. Valkonen

**Affiliations:** ^1^ Department of Agricultural Sciences University of Helsinki FI-00014 Helsinki Finland; ^2^ Department of Pathology, Division of Virology University of Cambridge Tennis Court Road Cambridge CB2 1QP UK; ^3^ Department of Plant Sciences University of Cambridge Downing Street Cambridge CB2 3EA UK; ^4^ International Potato Center Lima 12 Peru

**Keywords:** frameshift, gene expression, overlapping gene, RNA silencing suppression, RNA virus, transcriptional slippage

## Abstract

The single‐stranded, positive‐sense RNA genome of viruses in the genus *Potyvirus* encodes a large polyprotein that is cleaved to yield 10 mature proteins. The first three cleavage products are P1, HCpro and P3. An additional short open reading frame (ORF), called *pipo*, overlaps the P3 region of the polyprotein ORF. Four related potyviruses infecting sweet potato (*Ipomoea batatas*) are predicted to contain a third ORF, called *pispo*, which overlaps the 3′ third of the P1 region. Recently, *pipo* has been shown to be expressed via polymerase slippage at a conserved GA_6_ sequence. Here, we show that *pispo* is also expressed via polymerase slippage at a GA_6_ sequence, with higher slippage efficiency (∼5%) than at the *pipo* site (∼1%). Transient expression of recombinant P1 or the ‘transframe’ product, P1N‐PISPO, in *Nicotiana benthamiana* suppressed local RNA silencing (RNAi), but only P1N‐PISPO inhibited short‐distance movement of the silencing signal. These results reveal that polymerase slippage in potyviruses is not limited to *pipo* expression, but can be co‐opted for the evolution and expression of further novel gene products.

## Introduction

Several viruses of the genus *Potyvirus* (family *Potyviridae*) infect sweet potato (*Ipomoea batatas*). Currently, most fall within the monophyletic ‘*Sweet potato feathery mottle virus* (SPFMV) group’ (Untiveros *et al*., 2008). These include SPFMV, *Sweet potato virus C* (SPVC), *Sweet potato virus G* (SPVG) and *Sweet potato virus 2* (SPV2), but not *Sweet potato latent virus*. SPFMV is the most common virus infecting sweet potatoes worldwide. In mixed infections with *Sweet potato chlorotic stunt virus* (SPCSV; genus *Crinivirus*), it is associated with a severe sweet potato virus disease (SPVD), causing heavy yield losses (Gibson *et al*., 2004; Tairo *et al*., 2005). SPVD is particularly important in sub‐Saharan Africa, where sweet potato is the second most important root crop after cassava.

**Figure 1:**
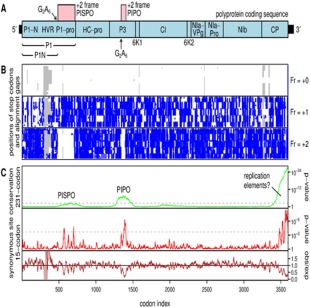
Genome organization of *Sweet potato feathery mottle virus* (SPFMV)‐group potyviruses. (a) Map of the ∼10.8‐kb genome showing the polyprotein open reading frame (ORF) (light blue) and overlapping *pipo* and *pispo* ORFs (pink). The P1‐N and P1‐pro domains and hypervariable region (HVR) are marked. For consistency with previous naming (SPFMV P1‐N and potyvirus P3N‐PIPO), we adopt the convention that ‘P1‐N’ refers to the N‐terminal domain, whereas ‘P1N’ refers to the entire region of P1 encoded upstream of the G_2_A_6_ slippage site. (b) Positions of stop codons (blue) in the three forward reading frames, and alignment gaps (grey) in an alignment of 31 SPFMV‐group sequences. (c) Conservation at synonymous sites in the alignment, using a 231‐codon sliding window (green) or a 15‐codon sliding window (red and brown). The upper panels (green, red) depict the probability that the degree of conservation within a given window could be obtained under a null model of neutral evolution at synonymous sites, whereas the bottom panel (brown) depicts the absolute amount of conservation as represented by the ratio of the observed number of substitutions within a given window to the number expected under the null model. The broken grey lines indicate a *P* = 0.05 threshold after applying a rough correction for multiple testing (namely 3519 codons/15‐ or 231‐codon window size).

The complete genome sequences of several SPFMV‐group viruses have been determined (Li *et al*., 2012; Pardina *et al*., 2012; Sakai *et al*., 1997; Untiveros *et al*., 2010; Yamasaki *et al*., 2010). Although all members of the genus *Potyvirus* encode a P1 protein, SPFMV‐group viruses encode the largest P1 among currently sequenced potyviruses (Fig. 1a) (Sakai *et al*., 1997; Valli *et al*., 2007). P1 is a serine protease that cleaves at its own C‐terminus, but is not known to be involved in other polyprotein‐processing events. Apart from its protease activity, the functions of P1 are not well established. It appears to play a variety of roles in different potyviruses, including enhancement of the RNA silencing suppression (RSS) activity of the helper component proteinase (HCpro), and roles in genome amplification and host specificity (reviewed in Rohožková and Navrátil, 2011; Valli *et al*., 2007). Although HCpro is generally considered to be the RSS protein in potyviruses (Anandalakshmi *et al*., 1998; Brigneti *et al*., 1998; Kasschau and Carrington, 1998), in members of some other potyvirid genera, P1 has been shown to be a potent RSS. In *Sweet potato mild mottle virus* (SPMMV; genus *Ipomovirus*), *Triticum mosaic virus* (genus *Poacevirus*) and *Wheat streak mosaic virus* (genus *Tritimovirus*), P1, but not HCpro, suppresses silencing (Giner *et al*., 2010; Tatineni *et al*., 2012; Young *et al*., 2012). *Cassava brown streak virus* (genus *Ipomovirus*) lacks HCpro, and P1 suppresses silencing (Mbanzibwa *et al*., 2009). *Cucumber vein yellowing virus* (genus *Ipomovirus*) also lacks HCpro, but has two P1 serine protease proteins, P1a and P1b, where P1b suppresses silencing (Valli *et al*., 2006). In *Potato virus Y* (PVY), P1 may be needed to stabilize the RSS protein HCpro (Tena Fernández *et al*., 2013). However, macluraviruses lack any P1 protein (Revers and Garcia, 2015).

The P1 protein of SPFMV‐group viruses appears to contain two distinct protein domains (Fig. 1a): the C‐terminal P1‐pro, which is homologous to the P1 serine protease encoded by all potyviruses, and an N‐terminal extension, designated P1‐N, which is absent from other potyviruses, but bears homology to the N‐terminal region of the P1 protein of the ipomovirus SPMMV (Untiveros *et al*., 2010; Valli *et al*., 2007). The P1‐N domain appears to have been transferred between ancestors of the two viruses, presumably during co‐infection of the common host. P1‐N and P1‐pro are separated by a hypervariable region (Untiveros *et al*., 2010). SPMMV P1‐N is an RSS which functions by binding Argonaute 1 (AGO1) (Giner *et al*., 2010). This is thought to be mediated via WG/GW motifs, and such motifs are a common feature of AGO‐binding proteins (Azevedo *et al*., 2010; El‐Shami *et al*., 2007; Giner *et al*., 2010; Till *et al*., 2007). Wild‐type SPFMV contains only one WG/GW motif in P1‐N, which was not found to confer any RSS activity (Szabó *et al*., 2012), whereas three WG/GW motifs are found in SPMMV P1‐N. Thus, despite the homology, it is unclear whether P1‐N serves a similar RSS function in SPFMV‐group viruses.

It was long believed that the potyvirus gene expression strategy was based on a large, single, open reading frame (ORF) encoding a polyprotein, which is processed to 10 mature proteins by three viral proteinases. However, Chung *et al*. (2008) discovered that there is another, short ORF in a different reading frame embedded within the region encoding P3. This ORF, designated *pipo* (‘pretty interesting *Potyviridae* ORF’), is conserved throughout the *Potyviridae* family. Polymerase slippage at a highly conserved GA_6_ sequence at the 5′ end of *pipo* results in the insertion of an extra A into 1%–2% of transcripts (Olspert *et al*., 2015; Rodamilans *et al*., 2015), leading, after translation and proteolytic cleavage, to the production of P3N‐PIPO, a ‘transframe fusion’ of the N‐terminal part of P3 (P3N) with the product (PIPO) of the *pipo* ORF. An additional, long alternative‐frame ORF in the region encoding P1‐pro was observed in the ‘SPFMV group’ of potyviruses and, in analogy with *pipo*, was named *pispo* (‘pretty interesting sweet potato potyvirus ORF’) (Fig. 1a) (Clark *et al*., 2012). The presence of multiple WG/GW motifs within the translation of *pispo* was noted by Clark *et al*. (2012), who suggested a potential role in RSS. Here, we present computational analyses of the *pispo* ORF that support its coding status, high‐throughput sequencing data demonstrating that *pispo* can be expressed as part of a transframe protein P1N‐PISPO via polymerase slippage at a conserved G_2_A_6_ slippery sequence, and experimental analyses that confirm that P1N‐PISPO is a potent suppressor of RNA silencing.

## Results

### Comparative genomic analysis of the overlapping *pispo* ORF

The polyprotein ORFs from the 28 full‐length SPFMV‐group sequences currently available in GenBank, plus three additional sequences determined in this study (see Experimental procedures), were extracted, translated, aligned as amino acid sequences and back‐translated to a nucleotide sequence alignment. Next, the positions of stop codons in the +1 and +2 reading frames relative to the polyprotein ORF, in all 31 sequences, were plotted. This confirmed the conserved presence of a long +2‐frame ORF overlapping the P1 region of the polyprotein ORF (Fig. 1b). The ORF has 230 codons in SPFMV and SPVC, and 228 codons in SPVG and SPV2 (measured from a ubiquitous 5′ G_2_A_6_ sequence; see below). The statistical significance of such a long ORF being conserved by chance over such a degree of phylogenetic divergence (estimated >900 phylogenetically independent substitutions within *pispo* in the SPFMV‐group sequence alignment) was assessed as described previously (Fang *et al*., 2012). Using this method, the probability of such a long +2 frame ORF occurring by chance at this location is *P* < 1 × 10^−16^.

Next, the alignment was analysed for enhanced conservation at polyprotein‐frame synonymous sites (an indicator of overlapping functional elements), as described previously (Firth, 2014). The analysis revealed regions of enhanced synonymous site conservation where the polyprotein ORF is overlapped by the *pipo* ORF (Chung *et al*., 2008), and at the 3′ end of the polyprotein ORF, where overlapping replicational RNA elements have been mapped in other potyvirus species (Fig. 1c) (Haldeman‐Cahill *et al*., 1998). Enhancement of polyprotein‐frame synonymous site conservation within the region overlapped by *pispo* was modest, suggesting that the PISPO amino acid sequence is not subject to strong purifying selection. Nonetheless, the mean rate of synonymous substitutions in the region of the polyprotein ORF overlapped by *pispo* was 10% below the genome average, with a corresponding total *P* value of 0.01 (Fig. 1c).

**Figure 2:**
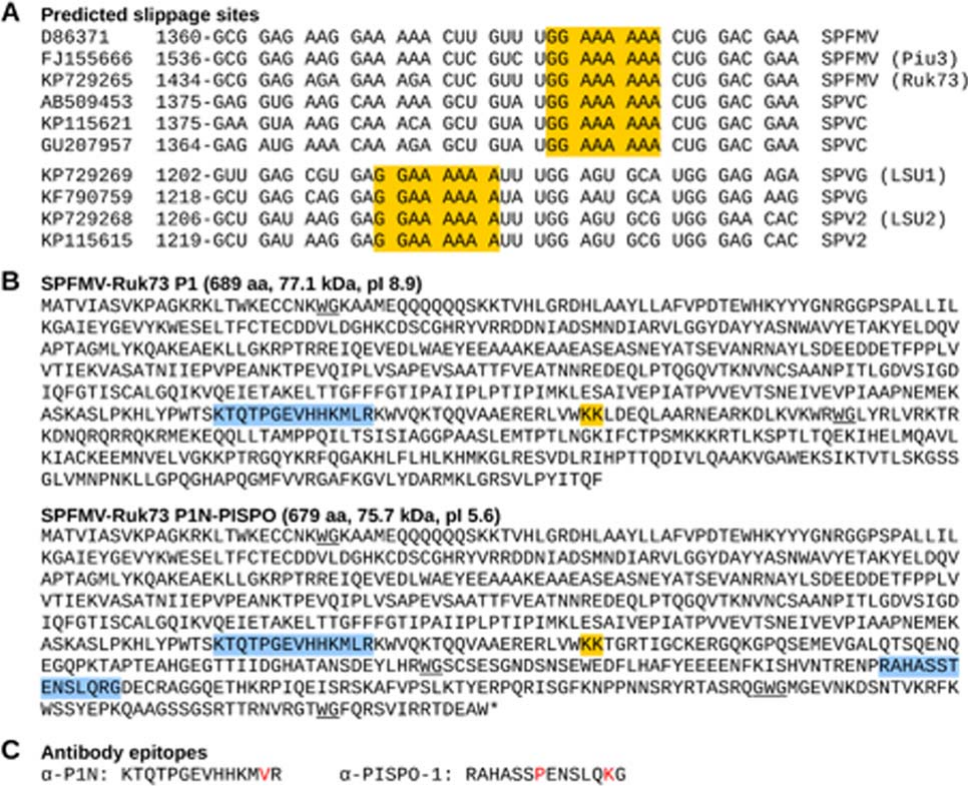
Representative nucleotide and peptide sequences. (a) Nucleotide sequences flanking the proposed slippage sites in representative *Sweet potato feathery mottle virus* (SPFMV)‐group sequences (GenBank accession numbers shown on left, species on right). Spaces separate polyprotein‐frame codons; *pispo* is in the +2 frame. The G_2_A_6_ sequence, where slippage is proposed to occur, is highlighted in orange. Numbers on the left indicate the genomic coordinates of the first nucleotide in each line. (b) Amino acid sequences of P1 and P1N‐PISPO in the Ruk73 isolate of SPFMV. The amino acids encoded at the slippage site are highlighted in orange. Sequences corresponding to the epitopes of the two polyclonal antibodies (pAbs) are highlighted in pale blue. GW/WG motifs are underlined. (c) Sequences of the epitopes against which the two pAbs were raised. Sequence differences from the Ruk73 isolate are highlighted in red. SPV2, *Sweet potato virus 2*; SPVC, *Sweet potato virus C*; SPVG, *Sweet potato virus G*.

At the 5′ end of the *pispo* ORF, there is a conserved G_2_A_6_ sequence (Figs 2a and S1, see Supporting Information). In SPFMV and SPVC isolates, this takes the form GG_AAA_AAA, where underscores separate polyprotein codons. In SPVG and SPV2 isolates, it takes the form G_GAA_AAA_A, displaced a few codons upstream from the sequence in SPFMV and SPVC (Fig. 2a). A similar highly conserved sequence, G_1–2_A_6–7_ (G_2_A_6_ in SPFMV‐group viruses), is present at the 5′ end of the *pipo* ORF in nearly all potyvirids (Chung *et al*., 2008). The presence of the same motifs at the 5′ end of *pispo* suggests that *pispo* is expressed by the same slippage mechanism as *pipo*. This would result in the production of a P1N‐PISPO fusion protein (Fig. 2b). In the Ruk73 strain of SPFMV, P1 has a predicted mass of 77.1 kDa, whereas P1N‐PISPO has a predicted mass of 75.7 kDa. Analysis of additional sequences with only partial coverage of the polyprotein ORF, but with coverage of the 5′ end of *pispo* and/or the entire *pispo* ORF, confirmed the conserved presence of the potential slippage site (14 of 14 sequences) and the conserved presence of the *pispo* ORF (nine of nine sequences).

### High‐throughput sequencing reveals polymerase slippage at the *pispo* and *pipo* G_2_A_6_ sites

**Figure 3:**
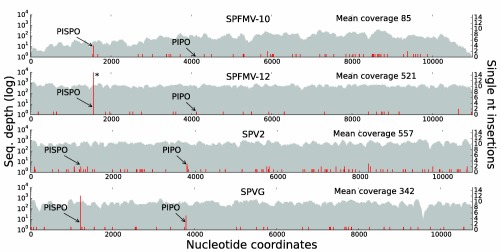
Single nucleotide insertions in short‐RNA sequencing of sweet potato virus‐infected plants. Short‐RNA reads from *Sweet potato virus 2* (SPV2), *Sweet potato virus G* (SPVG) and two *Sweet potato feathery mottle virus* (SPFMV) [10, sweet potato only infected with SPFMV; 12, sweet potato infected with SPFMV + *Sweet potato chlorotic stunt virus* (SPCSV)] samples were mapped to the corresponding reference genomes. For each sample, the sequencing depth of fully matched reads is plotted as a 100‐nucleotide moving average in grey (log scale on left axis). The mean coverage is indicated at the top right. Positions of single nucleotide insertions are indicated in red (linear scale on right axis). For each virus, the positions of the G_2_A_6_ sequences at the beginning of the *pispo* and *pipo* open reading frames (ORFs) are indicated with arrows. An asterisk (*) denotes the off‐scale value of 43 insertions at the *pispo* G_2_A_6_ site for sample SPFMV‐12.

The occurrence of the G_2_A_6_ sequence at the 5′ end of the *pispo* and *pipo* ORFs in different phases with respect to the polyprotein reading frame suggests that *pispo* and *pipo* are unlikely to be expressed via translational frameshifting, although, in principle, both are compatible with −1 ribosomal frameshifting (reviewed in Firth and Brierley, 2012). Further, using RNA‐folding software, such as alidot (Hofacker *et al*., 2002) and pknotsRG (Reeder *et al*., 2007), we failed to predict a convincing 3′ RNA secondary structure at a canonical spacing (i.e. 5–9 nucleotides) downstream of the slippery sites for the stimulation of −1 ribosomal frameshifting. To evaluate whether frameshifting could occur instead during RNA synthesis, we first utilized high‐throughput small RNA sequencing data obtained from SPFMV‐infected sweet potato plants and SPV2‐ and SPVG‐infected *Ipomoea setosa* plants. At both the *pispo* and *pipo* G_2_A_6_ sequences, a fraction of small RNA reads contained an additional ‘A’ insertion and, for most samples, insertions at these sites occurred well above background frequencies (Fig. 3; Table 1). In these data, ‘A’ insertions were found to occur at a frequency ranging from 0.9% to 11.4% at the *pispo* G_2_A_6_ site and 0.0% to 1.0% at the *pipo* G_2_A_6_ site, depending on the sample. The 0% values are probably a result of low coverage and the lower rate of insertions at the *pipo* site relative to the *pispo* site (see below). In comparison, average single nucleotide insertions at other sites across the genome were estimated to occur at a frequency ranging from 0.001% to 0.01% per nucleotide, depending on the sample (Table 1).

**Table 1:** Number of small RNA sequences with (FS) and without (WT) an ‘A’ insertion at the *pispo* or *pipo* G_2_A_6_ slippage sites.

Sample	PISPO			PIPO			Background % insertion rate
	WT	FS	FS %	WT	FS	FS %	
SPFMV‐10*	31	4	11.4	42	0	0.0	0.0106
SPFMV‐12*	576	43	7.0	219	1	0.5	0.0013
SPV2^†^	226	2	0.9	291	3	1.0	0.0035
SPVG^†^	219	12	5.2	580	5	0.9	0.0023

SPCSV, *Sweet potato chlorotic stunt virus*; SPFMV,*Sweet potato feathery mottle virus*; SPV2,*Sweet potato virus 2*; SPVG,*Sweet potato virus G*.

*10, sweet potato only infected with SPFMV; 12, sweet potato infected with SPFMV + SPCSV.

†*Ipomoea setosa* infected with SPV2 or SPVG.

**Figure 4:**
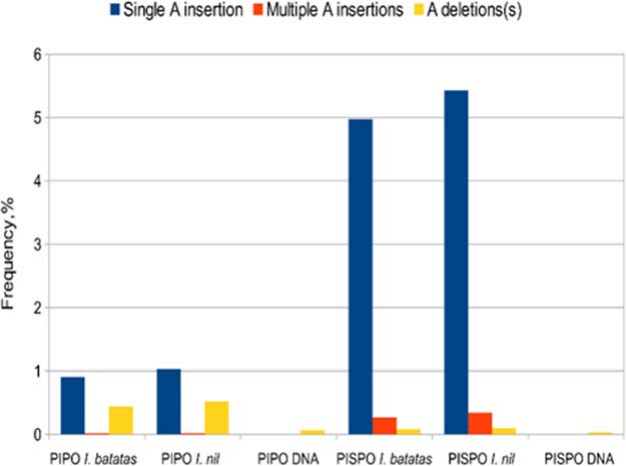
Transcriptional slippage at the *pispo* and *pipo* G_2_A_6_ sequences in *Sweet potato feathery mottle virus* (SPFMV)‐infected plants. Results from targeted high‐throughput sequencing of systemically infected *Ipomoea batatas* and *I. nil*. The frequencies of transcripts with a single ‘A’ insertion at the G_2_A_6_ sequence are shown in blue; the frequencies of transcripts with two or more inserted ‘A’ nucleotides are shown in orange; and the frequencies of transcripts with one or more ‘A’ nucleotides deleted are shown in yellow. Controls from plasmid template were included to assess the variability introduced during amplification and sequencing.

Although these results support the interpretation that polymerase slippage occurs at the *pispo* and *pipo* G_2_A_6_ sites, low coverage, variability between samples and low accuracy of insertion calling as a result of short read lengths mean that the slippage frequency is likely to be poorly estimated by this approach. Therefore, a separate sequencing experiment was conducted in which both the *pispo* and *pipo* sites in viral RNA were specifically targeted for amplification and sequencing. This experiment was conducted with RNA extracted from sweet potato or *I. nil* infected with SPFMV‐Ruk73, and led to sequencing depths ranging from 6.1 to 32.5 million reads for each slippage site region (Fig. 4; Table 2). Single ‘A’ insertions were observed within the *pispo* G_2_A_6_ sequence for 5.0% and 5.5% of sweet potato and *I. nil* reads, respectively. Within the *pipo* G_2_A_6_ sequence, single ‘A’ insertions were observed for 0.9% and 1.0% of reads, respectively. Control DNAs for the same regions showed no insertions at all in more than eight million reads, indicating that insertions did not occur during amplification, library preparation or sequencing. Parallel work with the *Turnip mosaic virus* G_2_A_6_ *pipo* slippage site indicated that any insertional slippage occurring during reverse transcription is less than ∼0.05% (Olspert *et al*., 2015). At a lower level, deletions were also observed to occur (Fig. 4), although these could be an artefact of deletional slippage during reverse transcription (Olspert *et al*., 2015).

**Table 2:** Targeted high‐throughput sequencing of the *pispo* and *pipo* G_2_A_6_ slippage sites.

Sample	# reads	Reads with a single ‘A’ inserted (i.e. G_2_A_7_)		Reads with >1 ‘A’ inserted		Reads with 1 or more ‘A's deleted	
		%	# reads	%	# reads	%	# reads
PIPO *Ipomoea batatas*	9149358	0.90	82637	0.02	1717	0.44	40314
PIPO *Ipomoea nil*	37396166	1.03	385513	0.02	8711	0.52	193787
PIPO DNA	8449879	0.00	0	0.00	0	0.07	5808
PISPO *I. batatas*	7298433	4.98	363120	0.27	19936*	0.08	5771
PISPO *I. nil*	30255818	5.45	1650021	0.31	95093^†^	0.10	28964
PISPO DNA	8142625	0.00	0	0.00	0	0.03	2366

*74% G_2_A_8_, 26% G_2_A_9._

†70% G_2_A_8_, 27% G_2_A_9_, 4% G_2_A_10._

### PISPO contains GW/WG motifs

The amino acid sequence of PISPO is less conserved than the amino acid sequence of the part of P1 that it overlaps (Table S1, see Supporting Information), e.g. there is 29%–40% amino acid identity in PISPO, but 48%–67% identity in P1‐pro between potyviruses that encode PISPO. Application of blastp, CS‐Blast, InterProScan, hhpred and phyre‐2 to PISPO revealed no significant homology to previously annotated or characterized proteins, as expected, as PISPO clearly evolved by ‘overprinting’ the ancestral P1‐pro domain (Altschul *et al*., 1990; Angermüller *et al*., 2012; Kelley and Sternberg, 2009; Quevillon *et al*., 2005; Söding *et al*., 2005). In addition, ∼60% of PISPO was predicted to be disordered according to PONDR‐FIT (Xue *et al*., 2010) (Fig. S2, see Supporting Information), again typical of many overlapping genes (Rancurel *et al*., 2009).

**Figure 5:**
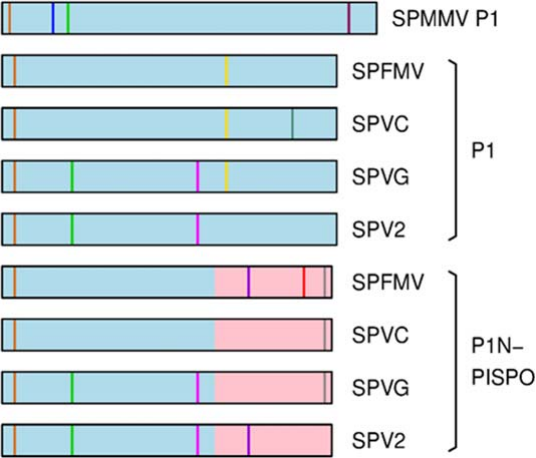
Locations of GW/WG sequences in P1 and P1N‐PISPO proteins of *Sweet potato feathery mottle virus* (SPFMV)‐group viruses. Schematic diagram of the P1 protein of *Sweet potato mild mottle virus* (SPMMV) and the P1 and P1N‐PISPO proteins of SPFMV‐group viruses, showing the positions of GW/WG sequences. The P1 sequence is indicated in light blue; the PISPO sequence is indicated in pink. GW/WG sequences are indicated by vertical coloured lines, with homologous instances (by blastp alignment) indicated with the same colour. SPV2, *Sweet potato virus 2*; SPVC, *Sweet potato virus C*; SPVG, *Sweet potato virus G*.

Further inspection of the amino acid sequence revealed WG/GW (‘AGO‐hook’) motifs. Such motifs play a key role within the P1‐N domain of SPMMV P1 (Giner *et al*., 2010) and some other plant viral silencing suppressors (Azevedo *et al*., 2010; de Ronde *et al*., 2014). Among 31 sequenced isolates, the PISPO sequences contain between one and three GW/WG motifs, whereas full‐length P1 and full‐length P1N‐PISPO contain between two and four GW/WG motifs (Fig. 5). It should be noted, however, that WG/GW di‐amino acids are not particularly uncommon in generic protein sequences.

### P1 and P1N‐PISPO are RNA silencing suppressors with distinguishable phenotypes

**Figure 6:**
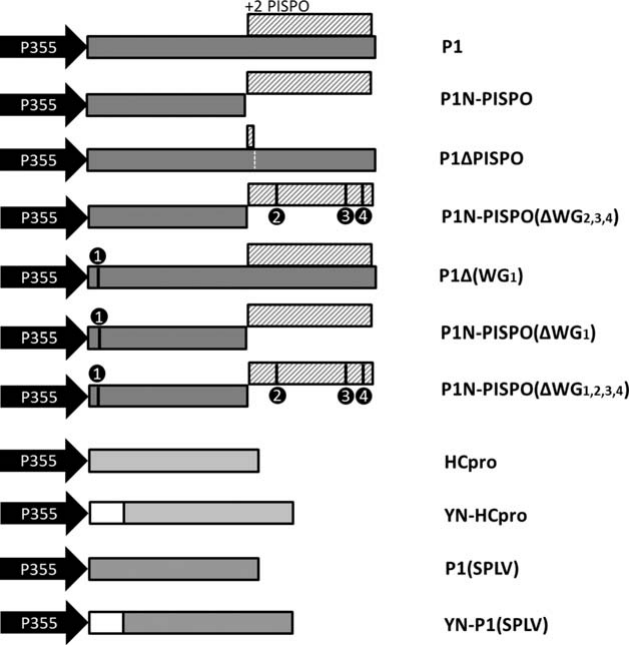
Binary vectors for the expression of viral proteins in leaves by agroinfiltration. *Cauliflower mosaic virus* 35S promoter‐driven binary vectors were designed to express the following proteins: P1, P1 of *Sweet potato feathery mottle virus* (SPFMV) [the shaded bar indicates the position of the +2 frameshift *pispo* open reading frame (ORF) encoding 230 residues]; P1N‐PISPO, engineered transframe sequence for the expression of the N‐proximal part of P1 together with PISPO; P1ΔPISPO, P1 including a stop codon inserted in the +2 frame *pispo* ORF to ensure that P1N‐PISPO is not produced; P1N‐PISPO(ΔWG2,3,4), P1N‐PISPO mutated at three positions (W_514_G_515_, W_624_G_625_ and W_665_G_666_; designated as WG motifs 2, 3 and 4, respectively) to substitute the tryptophan residues for alanines; P1(ΔWG_1_), P1 mutated to substitute residue W_25_ for alanine (WG motif 1); P1N‐PISPO(ΔWG_1_), P1N‐PISPO with W_25_ mutated to alanine; P1N‐PISPO(ΔWG1,2,3,4), P1N‐PISPO with all four WG motif tryptophans mutated to alanine; HCpro, HCpro of SPFMV; YN‐HCpro, the N‐proximal part of the yellow fluorescent protein (YN) fused to the N‐terminus of SPFMV HCpro; P1(SPLV), P1 of *Sweet potato latent virus*; YN‐P1(SPLV), the N‐proximal part of the yellow fluorescent protein (YN) fused to the N‐terminus of SPLV P1.

**Figure 7:**
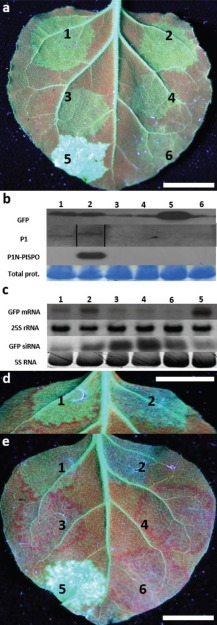
Suppression of sense‐mediated RNAi of green fluorescent protein (GFP) expression by potyviral proteins in leaves of GFP‐transgenic *Nicotiana benthamiana* line 16c. (a) Silencing of *gfp* was induced by overexpression of GFP mRNA ‘on the spot’ from a binary vector pBIN:GFP introduced by agroinfiltration, and interference with silencing was tested by co‐introduction of binary vectors for the expression of: (1) *Sweet potato feathery mottle virus* (SPFMV) P1; (2) SPFMV P1N‐PISPO; (3) SPFMV HCpro; (4) *Sweet potato latent virus* (SPLV) P1; (5) *Potato virus A* (PVA) HCpro; or (6) β‐glucuronidase (GUS) (control). The leaves were photographed at 4 days post‐infiltration (dpi) (a), 6 dpi (d) and 8 dpi (e). Scale bars indicate 2 cm. (b) Expressed proteins were detected at 4 dpi using antibodies to GFP, peptide antibodies generated against an epitope common to P1 and P1N‐PISPO, and peptide antibodies specific to the PISPO domain of P1N‐PISPO. In the P1 panel, lane 2 was loaded in a different order in the gel, and spliced electronically afterwards (indicated by black vertical lines), for consistency with other panels. Staining of total proteins by Coomassie blue was used as a loading control (shown for GFP gel). (c) Northern blot analysis for the detection of GFP mRNA and *gfp*‐derived small interfering (si)RNA in leaf tissues co‐infiltrated with pBIN:GFP and the viral constructs in (A) at 4 dpi; *gfp* was used as a probe and 28S and 5S ribosomal RNAs (rRNA) were used as loading controls. Note the different order of samples 5 and 6 in panels (b) and (d). (d) The infiltrated leaf tissue expressing P1 was surrounded by a red halo (left), in contrast with the tissue expressing P1N‐PISPO (right), which was most apparent at 6 dpi. (e) Suppression of silencing by P1, P1N‐PISPO and PVA HCpro was indicated by GFP fluorescence in the co‐infiltrated leaf tissue at 8 dpi, whereas no suppression of silencing was observed with SPFMV HCpro and SPLV P1.

To test whether P1 and/or P1N‐PISPO have RSS activity, we used a standard silencing assay, whereby transgenic *Nicotiana benthamiana* line 16c plants that constitutively express green fluorescent protein (GFP) were agroinfiltrated with recombinant *Agrobacterium tumefaciens* strains harbouring binary expression constructs for GFP (pBIN:GFP) and a test protein (e.g. pBIN:P1). Overexpression of GFP mRNA from pBIN:GFP induces sense‐mediated silencing of GFP and results eventually in a red leaf patch under UV light, unless the test protein suppresses silencing, in which case the leaf patch will show enhanced green fluorescence under UV light. We tested various potyvirus proteins in this system (Fig. 6). At 4 days post‐infiltration (dpi), tissues co‐infiltrated for expression of GFP and P1 or P1N‐PISPO showed slightly brighter GFP fluorescence than those infiltrated for expression of SPFMV HCpro, *Sweet potato latent virus* (SPLV) P1 or a β‐glucuronidase (GUS) control (Fig. 7a), indicating that P1 and P1N‐PISPO have RSS activity. Leaf tissues expressing *Potato virus A* (PVA) HCpro exhibited the strongest GFP signals (Fig. 7a). These phenotypic differences correlated positively with the accumulation of GFP (Fig. 7b) and GFP mRNA (Fig. 7c), and were negatively correlated with the accumulation of GFP mRNA‐derived small interfering RNA (siRNA) in the leaf tissue (Fig. 7c). The expression of P1 and P1N‐PISPO was verified by western analysis using peptide antibodies (Abs) generated against an epitope (Fig. 2c) common to P1 and P1N‐PISPO (blot ‘P1’ in Fig. 7b) and peptide Abs specific to P1N‐PISPO (Fig. 7b). The observed bands were of slightly different sizes than expected, possibly as a result of post‐translational modifications.

The suppression of silencing by P1 and P1N‐PISPO was quite apparent at 6 dpi (Fig. 7d) and at 8 dpi (Fig. 7e), as indicated by GFP fluorescence in the co‐infiltrated leaf tissue, whereas the leaf spots infiltrated for the expression of SPFMV HCpro, SPLV P1 and GUS had lost GFP fluorescence (Fig. 7e). However, P1 and PIN‐PISPO differed in that a red halo developed around the leaf tissue infiltrated for the expression of P1. The red halo was most apparent at 6 dpi (Fig. 7d,e, leaf spot 1), indicating short‐distance movement of the silencing signal. A red halo also developed around the leaf spots infiltrated for the expression of SPFMV HCpro, SPLV P1 and GUS (Fig. 7e). In contrast, only a faint red halo, if any, developed around the leaf tissue infiltrated for the expression of P1N‐PISPO (Fig. 7d,e, leaf spot 2), indicating interference with the short‐distance signalling for silencing.

As no Abs were available for detection of SPFMV HCpro and SPLV P1, these proteins were also expressed tagged with the N‐proximal part (YN) of yellow fluorescent protein (YFP) (Fig. 6) and tested by western blot using anti‐YN Abs. YN‐P1 (SPLV) was detected at 2 dpi (Fig. S3, see Supporting Information), but only occasionally at later time points (3–5 dpi), whereas YN‐HCpro could not be detected at any time point (2–10 dpi) in eight independent experiments.

**Figure 8:**
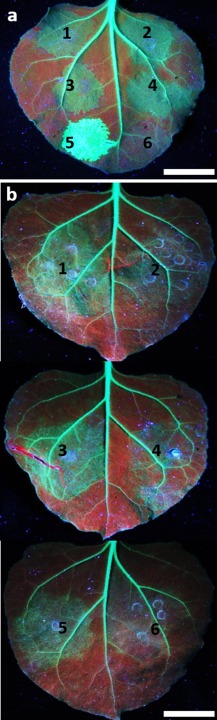
Effects of mutation of the WG motifs in P1 and P1N‐PISPO on the suppression of sense‐mediated RNAi of green fluorescent protein (GFP) expression in leaves of GFP‐transgenic *Nicotiana benthamiana* line 16c. Silencing of *gfp* was induced by overexpression of GFP mRNA from a binary vector pBIN:GFP introduced by agroinfiltration, and interference with silencing was tested by co‐introduction of binary vectors for expression of *Sweet potato feathery mottle virus* (SPFMV) P1, P1N‐PISPO or their mutated forms (see Fig. 3). (a) Suppression of silencing by: (1) P1, (2) P1N‐PISPO, (3) P1ΔPISPO, (4) P1N‐PISPO(ΔWG_2,3,4_), (5) *Potato virus A* (PVA) HCpro or (6) β‐glucuronidase (GUS) control at 4 days post‐infiltration (dpi). (b) Half‐agroinfiltrated leaf arrays were used to test the suppression of silencing by: (1) P1, (2) P1(ΔWG_1_), (3) P1N‐PISPO, (4) P1N‐PISPO(ΔWG_1_), (5) P1N‐PISPO(ΔWG_2,3,4_) or (6) P1N‐PISPO(ΔWG_1,2,3,4_). Scale bars indicate 2 cm.

As it is possible that the P1 construct could also express P1N‐PISPO via translational or transcriptional frameshifting, we generated another P1 expression construct, P1ΔPISPO, in which expression of the *pispo* ORF was prevented by the insertion of a stop codon (Fig. 6). As with P1 (leaf spot 1; Fig. 8a), P1ΔPISPO (leaf spot 3; Fig. 8a) suppressed GFP silencing and was associated with the formation of a red halo.

### The WG motif at the N‐terminus of P1 is pivotal for suppression of RNA silencing

The completely conserved WG motif near the N‐terminus of SPFMV‐group P1 (residues 25–26) aligns with the first WG motif in SPMMV P1 (Fig. 5), which has been shown to be required for SPMMV P1 RSS activity (Giner *et al*., 2010). To test whether this WG motif is also required for the RSS activity of SPFMV P1 and/or P1N‐PISPO, we mutated the tryptophan residue (W_25_) to alanine and tested the mutated P1 and P1N‐PISPO for RSS activity, as above. In contrast with P1 and P1N‐PISPO (Fig. 8b, leaf spots 1 and 3, respectively), the mutated constructs for P1(ΔWG_1_) and P1N‐PISPO(ΔWG_1_) (Fig. 6) were unable to suppress silencing (Fig. 8b, leaf spots 2 and 4, respectively). Similarly, when all four WG motifs were mutated [P1N‐PISPO(ΔWG_1,2,3,4_)] (Fig. 6), no suppression of silencing was observed (Fig. 8b, leaf spot 6). In contrast, similar mutations introduced in all three WG motifs in the PISPO domain [P1N‐PISPO(ΔWG_2,3,4_)] (Fig. 6) did not prevent the suppression of silencing (Fig. 8b, leaf spot 5). Taken together, these results indicate that the WG motif near the N‐terminus of P1 is essential for suppression of RNAi by both P1 and P1N‐PISPO.

## Discussion

Potyviruses were believed to use a single polyprotein gene expression strategy until the recent discovery of the overlapping *pipo* ORF (Fig. 1a) (Chung *et al*., 2008). The *pipo* ORF has a highly conserved G_1–2_A_6–7_ sequence at its 5′ end. Previously, it was proposed that the expression of P3N‐PIPO might depend on translational or transcriptional frameshifting occurring at this site (Chung *et al*., 2008). However, the true nature of the expression mechanism was not confirmed until recently, when, during the preparation of this article, both we and others observed the production of virus transcripts with single nucleotide insertions at the *pipo* G_1–2_A_6–7_ sequence in various potyviruses, thus allowing the expression of P3N‐PIPO (this study; Olspert *et al*., 2015; Rodamilans *et al*., 2015). The ubiquitous presence of a G_2_A_6_ sequence at the 5′ end of the *pispo* ORF, conserved in all SPFMV‐group sequences, suggested that *pispo* might be expressed via the same polymerase slippage mechanism, leading to a P1N‐PISPO trans‐frame fusion protein. The potential for P1N‐PISPO to be expressed in this way was confirmed by the observation of edited transcripts in our small RNA sequencing data for SPFMV, SPV2 and SPVG, and our targeted high‐throughput sequencing of SPFMV RNA. The latter data also indicate that slippage at the SPFMV *pispo* slip site (5.0%–5.4%) is significantly more efficient than at the SPFMV *pipo* slip site (0.9%–1.0%). Whilst this article was in preparation, a similar difference was also observed by others (Rodamilans *et al*., 2015). The reasons for the differences between the two sites are unknown, but may be a result of the flanking nucleotide context which may affect the stability of the template:nascent RNA duplex adjacent to the slippage site (Hausmann *et al*., 1999). Although we have currently been unable to detect P1N‐PISPO in infected plants by western analysis (perhaps because of low expression, masking of the Ab epitope and/or the kinetics of virus infection and protein turnover), the presence of edited transcripts implies that P1N‐PISPO can indeed be expressed during virus infection.

P1 is the most variable potyvirus protein in terms of length and sequence (Adams *et al*., 2005; Valli *et al*., 2007). The discovery of PISPO adds a new dimension to the functional variability occurring in the P1 region. Unlike PIPO, which is found universally in the *Potyviridae* family, PISPO is restricted to potyviruses in the SPFMV group. It does not occur in the related SPLV, nor in the ipomovirus SPMMV with which SPFMV‐group viruses share a related P1‐N domain. The PISPO sequence is highly variable (Table S1) (Li *et al*., 2012) and less conserved than the overlapping region of P1‐pro, in clear contrast with PIPO and the region of P3 that it overlaps (Chung *et al*., 2008). Differences in the evolutionary plasticity of *pipo* and *pispo* may reflect the functional importance of the corresponding transframe proteins. P3N‐PIPO is an essential protein, and appears to be of ancient origin, as attested by its conservation throughout the *Potyviridae* family, whereas P1N‐PISPO seems to be a more recent evolutionary development and may therefore not yet have acquired a critical role, allowing more sequence plasticity, as has been observed for other recently evolved overlapping viral genes (Sabath *et al*., 2012). PISPO is predicted to be largely disordered (Fig. S2). The function of such an intrinsically unstructured protein region may be largely independent of most of the primary sequence, allowing for rapid evolution and exploration of sequence space (Gitlin *et al*., 2014).

Although it is clear that PISPO is biologically relevant, as an ORF of this length would not be conserved by chance (*P* < 1 × 10^−16^) over the given phylogenetic divergence, at present there is no infectious clone of SPFMV, and so it is not possible to verify the functional relevance of P1N‐PISPO during infection. However, given the importance of WG/GW domains in other silencing suppressors (Giner *et al*., 2010; de Ronde *et al*., 2014), the evaluation of P1N‐PISPO for RSS activity was an obvious course. Surprisingly, we found that *both* P1N‐PISPO *and* P1 are RNA silencing suppressors in SPFMV. Intrinsic silencing suppression has not been described previously for P1 proteins of potyviruses (Rajamäki *et al*., 2005; Tena Fernández *et al*., 2013), nor was it found herein for P1 of SPLV (which clusters as an immediate outgroup to the SPFMV group). Most recent reports assign potyvirus P1 a role for increasing the accumulation of HCpro and consequently enhancing its silencing activity (Tena Fernández *et al*., 2013). We also showed that P1 and P1N‐PISPO seem to have different silencing activities in SPFMV, a strategy described for different RSS proteins of other viruses as well (Lu *et al*., 2004; Senshu *et al*., 2011). P1 operates only at a single‐cell or local level, as evidenced by the formation of the red halo around the patch (Fig. 7), as also reported for SPMMV P1 and a few other virus silencing suppressors (Baumberger *et al*., 2007; Giner *et al*., 2010). The effect of P1 on silencing was a result of the sole action of this protein, and participation of traces of P1N‐PISPO can be discarded as the phenotype was identical to P1ΔPISPO (Fig. 8), in which PISPO is made untranslatable by introducing a stop codon. However, P1N‐PISPO transiently inhibited RNA silencing at both local and short‐range levels, as reported for suppressors that sequester siRNA, including PVA HCpro and tombusvirus P19 (Lakatos *et al*., 2004; Shiboleth *et al*., 2007). The elimination of the three PISPO WG/GW motifs did not remove the suppressor capacity of P1N‐PISPO (Fig. 8). However, the elimination of the single WG near the N‐terminus of P1‐N completely eliminated RSS activity of both P1 and P1N‐PISPO. Thus, the differing C‐termini of P1 and P1N‐PISPO modulate the RSS activity resident within P1‐N.

The results of our experiments differ from those of a previous study in which P1 of SPFMV was reported not to suppress RNA silencing (Szabó *et al*., 2012), probably because a different experimental system and SPFMV isolate were used.

The functional characterization of P1 and P1N‐PISPO requires further experimental work, in particular during SPCSV synergisms. SPCSV RNase3 protein mediates these synergisms through the suppression of antiviral defence in sweet potato plants, allowing potyviruses to enhance titres (Cuellar *et al*., 2009). Nevertheless, symptom severity, even among SPFMV‐group viruses, varies greatly (Gutierrez *et al*., 2003; Kokkinos and Clark, 2006; Untiveros *et al*., 2007), and this is not necessarily related to the enhancement of viral replication (Kokkinos and Clark, 2006). This suggests that additional viral determinants may be required for the development of unusually severe disease in sweet potato plants co‐infected with SPFMV and SPCSV. Such determinants might include P1 and P1N‐PISPO. Therefore, the results of this study may have important implications for understanding the devastating synergistic diseases caused by these viruses in sweet potato.

## Experimental Procedures

### Viruses

SPFMV isolate Ruk73 from Uganda (Tugume *et al*., 2010) was used to generate all the SPFMV‐derived expression vectors. In addition, a Taiwanese isolate of SPLV (Wang *et al*., 2013), available from the International Potato Center (CIP) reference virus collection, was used to generate the corresponding P1 expression construct. SPFMV‐Piu3 (Kreuze *et al*., 2009), and SPVG‐LSU1 and SPV2‐LSU2 (kindly provided by Dr Christopher A. Clark, Louisiana State University, Baton Rouge, LA, USA), available from the CIP reference isolate collection, were used for small RNA sequencing.

### Sequencing of SPVG and SPV2 by assembly of siRNAs

siRNA sequencing (provider: Fasteris Life Sciences SA, Plan‐les‐Ouates, Switzerland) and assembly were performed on leaf samples from *I. setosa* plants infected with SPVG‐LSU1 or SPV2‐LSU2, as described by Kreuze *et al*. (2009). Original siRNA reads can be found at: https://research.cip.cgiar.org/confluence/display/cpx/CIP.sweetpotato.2015. The full genome sequences of SPVG (10 771 nucleotides) and SPV2 (10 732 nucleotides) were assembled at an average sequencing depth of 342‐ and 557‐fold, respectively, and deposited in GenBank (Table S2, see Supporting Information).

### Computational analysis

The complete genomes of SPFMV‐Ruk73, SPVG‐LSU1 and SPV2‐LSU2 were determined in this study (see above). Additional SPFMV‐group nucleotide sequences in GenBank were identified using tblastn (Altschul *et al*., 1990) (see Table S2 for accession numbers). Coding sequences were extracted, translated, aligned and back‐translated to produce nucleotide sequence alignments using EMBOSS and ClustalW (Larkin *et al*., 2007; Rice *et al*., 2000). Synonymous site conservation was calculated as described previously (Firth, 2014). The probability of the conserved absence of stop codons in the *pispo* ORF occurring by chance (i.e. if *pispo* was actually non‐coding) was assessed via P1‐frame alignment codon column shuffling, as described previously (Fang *et al*., 2012).

### Plasmids

A schematic diagram of the vectors used in this study is displayed in Fig. 6. Briefly, DNA fragments containing the complete coding regions of SPFMV P1 and HCpro (isolate Ruk73), and SPLV P1 (KC443039.1), were amplified by polymerase chain reaction (PCR) using Phusion high‐fidelity DNA polymerase (Thermo Scientific, Waltham, MA, USA) and cloned into the pBIN61 binary vector using *Xba*I/*Xma*I restriction sites. The sequence encoding the YFP N‐proximal fragment (YN) was added to the 3′ end of SPFMV HCpro and SPLV P1 using a similar approach to that described previously (Haikonen *et al*., 2013). Appropriate primers were used to introduce an AUG start or a UAA stop codon for the cloned regions where required. Thus, binary vectors containing wild‐type viral proteins included pBIN61:SPFMV‐P1, pBIN61:YN‐SPFMV‐HCpro and pBIN61:YN‐SPLV‐P1.

In addition, SPFMV P1 (hereafter called P1) was cloned into the expression vector pQE 30 (Qiagen, Venlo, the Netherlands) using *Hind*III/*Bam*HI restriction sites, generating plasmid pQE30:P1. From this plasmid, other constructs were developed, including P1N‐PISPO, P1ΔPISPO, P1N‐PISPO(ΔWG_2,3,4_), P1(ΔWG_1_), P1N‐PISPO(ΔWG_1_) and P1N‐PISPO(ΔWG_1,2,3,4_), using appropriate primers and the Site Directed Mutagenesis Kit (Stratagene, La Jolla, CA, USA). The transframe P1N‐PISPO construct contains a 1‐nucleotide ‘A’ insertion at site 1464 (i.e. G_2_A_6_ to G_2_A_7_ mutation), resulting in a frame change and the production of a P1N‐PISPO protein identical to that expected from polymerase slippage. In addition, two silent ‘A’ to ‘G’ mutations were introduced in P1N‐PISPO at genomic sites 1460 and 1463 within the G_2_A_7_ sequence (Lys 448 and 449; GG_AAA_AAA_A to GG_AAG_AAG_A) in order to prevent translational or transcriptional slippage occurring on the run of seven 'A's. P1ΔPISPO is a modified version of P1 including a synonymous mutation (GCA to GCT) at serine‐456 in the polyprotein frame, resulting in a +2 frame stop codon that prevents any accidental expression of P1N‐PISPO from this construct. P1N‐PISPO(ΔWG_2,3,4_) has the tryptophan residue in each of the three WG motifs in P1N‐PISPO replaced with alanine. PCR‐derived DNA fragments from the mutant versions were subsequently cloned into pBIN61, generating the corresponding plasmids pBIN61:P1N‐PISPO, pBIN61:P1ΔPISPO, pBIN61:P1N‐PISPO(ΔWG_2,3,4_), pBIN61:P1(ΔWG_1_), pBIN61:P1N‐PISPO(ΔWG_1_) and pBIN61:P1N‐PISPO(ΔWG_1,2,3,4_). All plasmids were verified by sequencing. The primers used are shown in Table S3 (see Supporting Information). Additional binary vectors pAHCpro, p35S‐GUS‐INT and pAGFP, used in the present study for the expression of PVA HCpro, GUS‐INT and GFP, respectively, have been described previously (Kreuze *et al*., 2005).

### Agroinfiltration assays

Agroinfiltration was carried out as described in Johansen and Carrington (2001). *Agrobacterium tumefaciens* C58C1 strains carrying the indicated plasmids were combined before infiltration. The different *Agrobacterium* cultures were diluted with infiltration medium so that equal final optical densities were reached before combining cultures in a 1 : 1 ratio for infiltration. Transgenic *N. benthamiana* line 16c, which expresses GFP, was used for infiltration (seeds kindly provided by Professor D. Baulcombe, Department of Plant Sciences, University of Cambridge, Cambridge, UK). Co‐infiltration was carried out with two *A. tumefaciens* strains expressing GFP and P1, P1N‐PISPO, SPFMV HCpro, SPLV P1, PVA HCpro, GUS, P1ΔPISPO, P1N‐PISPO(ΔWG_2,3,4_), P1(ΔWG_1_), P1N‐PISPO(ΔWG_1_) or P1N‐PISPO(ΔWG_1,2,3,4_). GFP fluorescence was monitored daily by epi‐illumination with a hand‐held UV source (Blackray UVP 100 AP, Thermo Scientific). Photographs were taken with a digital camera, and images were prepared with Photoshop software (Adobe, San Jose, CA, USA). Samples were collected at different time points to ensure the detection of the relevant proteins.

### Antibodies and western blotting

Polyclonal Abs to predicted antigens within PISPO and P1N (present in both P1 and P1N‐PISPO) were prepared by GenScript (Piscataway, NJ, USA). Anti‐P1N was raised against peptide sequence KTQTPGEVHHKMVR. Anti‐PISPO was raised against peptide sequence RAHASSPENSLQKG. Rabbits were injected with one of the two peptides, and Abs were affinity purified from immune sera. The detection of GFP YN‐tagged proteins and GFP was achieved using GFP‐specific polyclonal rabbit antiserum (Invitrogen, Carlsbad, CA, USA). Total proteins were extracted from leaves as follows: 20 mg of leaf tissue were homogenized in liquid nitrogen and resuspended in 80 μL of 2 × extraction buffer [0.1 m Tris‐HCl, 20% glycerol, 10% (v/v) 2β‐mercaptoethanol, 4% (w/v) sodium dodecyl sulfate (SDS), 0.2% bromophenol blue], boiled for 5 min and clarified by centrifugation. When needed, proteins were concentrated from larger amounts of plant material using acetone precipitation. Proteins were analysed on a 12% SDS‐polyacrylamide gel by electrophoresis and electroblotted onto a Hybond‐P polyvinylidene fluoride membrane (GE Healthcare, Chalfont St Giles, Buckinghamshire, UK). Membranes were blocked in 50 mL of Tris‐HCl (TBS) with 0.1% Tween 20 and 5% skimmed milk powder, and then probed with the corresponding Abs (1 : 5000 P1, PISPO) at 4°C overnight. Following washing with 1 × TBS containing 0.1% Tween 20, the membrane was incubated with polyclonal donkey anti‐rabbit immunoglobulins (IgGs) conjugated with horseradish peroxidase (HRP) (GE Healthcare). Proteins were detected with the enhanced chemiluminescence method using Super Signal West Femto chemiluminescent substrate (Thermo Scientific), and visualized by exposure to photographic film (Kodak, Rochester, NY, USA).

### RNA analysis

The accumulation of GFP mRNA and GFP mRNA‐derived siRNA was evaluated by northern blot analysis in leaf tissues agroinfiltrated for co‐expression of GFP and P1, P1N‐PISPO, SPFMV HCpro, SPLV P1, PVA HCpro or GUS. Total RNA was extracted with TRIzol LS Reagent (Invitrogen) according to the manufacturer's recommendations and low‐molecular‐weight (LMW) and high‐molecular‐weight (HMW) RNA fractions were separated as described in Cuellar *et al*. (2009). LMW RNA (15 μg/lane) and HMW RNA (10 μg/lane) were separated by electrophoresis in a denaturing 15% polyacrylamide gel and 1.2% (w/v) agarose gel, respectively, both containing formaldehyde. The two gels were transferred to a nylon membrane (GE Healthcare) and cross‐linked chemically with 1‐ethyl‐3‐(3‐dimethylaminopropyl)carbodi‐imide (Pall and Hamilton, 2008) or by UV light (Sambrook and Russell, 2001), respectively. A GFP mRNA‐specific radioactive RNA probe was synthesized, fractionated with carbonate hydrolysis and used to probe the membranes for GFP mRNA and GFP mRNA‐derived siRNA, as described previously (Cuellar *et al*., 2009). Signals were detected by autoradiography on an X‐ray film (Kodak).

### High‐throughput sequencing

Targeted high‐throughput sequencing was performed on total RNA extracted from SPFMV‐Ruk73‐infected sweet potato or *I. nil* plants, as described in Olspert *et al*. (2015), except that reverse transcription‐polymerase chain reaction (RT‐PCR) was performed with primers to amplify the regions surrounding the putative slippage sites for *pispo* (target site, excluding primer and adaptor sequences: ACTCGTTTGGAAAAAACTGGAC, corresponding to SPFMV‐Ruk73 nucleotides 1448–1469) and *pipo* (target site, excluding primer and adaptor sequences: CTCATGGAAAAAATTTGGGAT, corresponding to SPFMV‐Ruk73 nucleotides 4017–4037)

High‐throughput sequencing of small RNA was performed on RNA extracted from infected sweet potato (SPFMV‐Piu3) or *I. setosa* (SPVG‐LSU1 and SPV2‐LSU2) plants as described above. Short‐RNA reads, both forward and reverse sense, were mapped to the respective reference genomes using BWA (Li and Durbin, 2009), and positions of insertions were determined from the BWA CIGAR and TAG report fields. Initial background single nucleotide insertion rates were calculated by dividing the observed number of insertions (excluding those at the *pispo* and *pipo* G_2_A_6_ sites) by the product of the mean read depth and genome length. As a result of the short length of reads (21–24 nucleotides) and BWA not reporting insertions or deletions (indels) that occur within 5 nucleotides of the termini of reads, these initial values were adjusted for the potentially unreported indels by multiplying by the correction factor ∑*_i_ P_i_ i*/(*i* – 10), where *i* ranges over the read lengths (i.e. 21–24 nucleotides), *P_i_* is the fraction of mapped reads that have length *i*, and *i*/(*i* – 10) is the reciprocal of the proportion of insertions occurring within a read of length *i* that would be detected by BWA (i.e. are not within 5 nucleotides of the read termini).

### Accession numbers

GenBank sequence accession numbers are provided in Table S2.

## Supporting information

Additional Supporting Information may be found in the online version of this article at the publisher's website:

**Fig. S1** Predicted *pispo* G_2_A_6_ slippage sites. Predicted *pispo* G_2_A_6_ slippage sites (orange highlighted) in all available full‐length *Sweet potato feathery mottle virus* (SPFMV)‐group sequences. SPV2, *Sweet potato virus 2*; SPVC, *Sweet potato virus C*; SPVG, *Sweet potato virus G*.

**Fig. S2** PONDR® disorder predictions for P1 and P1N‐PISPO. Common regions predicted to be unstructured or disordered include a small region of ∼25 residues within the N‐terminus of P1N, and the hypervariable region (residues 200–300). In P1, a third region of predicted disorder occurs around residues 459–522, whereas, in P1N‐PISPO, a third region of predicted disorder encompasses almost the entire PISPO domain starting around residue 444. Predicted non‐disordered domains have scores no higher than 0.5.

**Fig. S3** Expression and detection of YN‐P1(SPLV) in agroinfiltrated leaf tissue of green fluorescent protein (GFP)‐transgenic *Nicotiana benthamiana* line 16c. (a) Neither (1) YN‐P1(SPLV) (left half of the leaf) nor (2) YN‐HCpro (right half of the leaf) of *Sweet potato feathery mottle virus* (SPFMV) suppressed *gfp* silencing and no enhancement of GFP fluorescence was observed at 4 days post‐infiltration (dpi). The infiltrated tissue was surrounded by a red edge indicating short‐distance movement of the silencing signal. Scale bar indicates 2 cm. (b) Expressed YN‐P1(SPLV) protein was detected at 2 dpi using anti‐YN antibodies, but not at 3, 4 or 5 dpi. The upper band indicated with an arrow (2d) corresponds to the expected size (72 kDa) of YN‐P1(SPLV). The small arrow on the left indicates the position of the 70‐kDa protein marker. Staining of total proteins by Coomassie blue was used as a loading control.

**Table S1** Mean amino acid inter‐ and intra‐species identities. Identities (%) calculated for the PISPO (*italics*; upper left) and P1‐pro (roman; lower right) domains.

**Table S2** Virus species and accession number for sequences used for the computational analysis of the *pispo* open reading frame (ORF). Sequences of *Sweet potato virus G* (SPVG)‐LSU1 and *Sweet potato virus 2* (SPV2)‐LSU2 were determined in this study, and partial sequences with coverage of *pispo* also included are indicated in a separate column.

**Table S3** Primer sequences used in this study.

## References

[mpp12366-bib-0001] Adams, M. , Antoniw, J. and Fauquet, C. (2005) Molecular criteria for genus and species discrimination within the family Potyviridae. Arch. Virol. 150, 459–479. 15592889 10.1007/s00705-004-0440-6

[mpp12366-bib-0002] Altschul, S.F. , Gish, W. , Miller, W. , Myers, E.W. and Lipman, D.J. (1990) Basic local alignment search tool. J. Mol. Biol. 215, 403–410. 2231712 10.1016/S0022-2836(05)80360-2

[mpp12366-bib-0003] Anandalakshmi, R. , Pruss, G.J. , Ge, X. , Marathe, R. , Mallory, A.C. , Smith, T.H. and Vance, V.B. (1998) A viral suppressor of gene silencing in plants. Proc. Natl. Acad. Sci. USA, 95, 13 079–13 084. 9789044 10.1073/pnas.95.22.13079 PMC23715

[mpp12366-bib-0004] Angermüller, C. , Biegert, A. and Söding, J. (2012) Discriminative modelling of context‐specific amino acid substitution probabilities. Bioinformatics, 28, 3240–3247. 23080114 10.1093/bioinformatics/bts622

[mpp12366-bib-0005] Azevedo, J. , Garcia, D. , Pontier, D. , Ohnesorge, S. , Yu, A. , Garcia, S. , Braun, L. , Bergdoll, M. , Hakimi, M.A. and Lagrange, T. (2010) Argonaute quenching and global changes in Dicer homeostasis caused by a pathogen‐encoded GW repeat protein. Genes Dev. 24, 904–915. 20439431 10.1101/gad.1908710 PMC2861190

[mpp12366-bib-0006] Baumberger, N. , Tsai, C.‐H. , Lie, M. , Havecker, E. and Baulcombe, D.C. (2007) The Polerovirus silencing suppressor P0 targets ARGONAUTE proteins for degradation. Curr. Biol. 17, 1609–1614. 17869110 10.1016/j.cub.2007.08.039

[mpp12366-bib-0007] Brigneti, G. , Voinnet, O. , Li, W.X. , Ji, L.H. , Ding, S.W. and Baulcombe, D.C. (1998) Viral pathogenicity determinants are suppressors of transgene silencing in *Nicotiana benthamiana* . EMBO J. 17, 6739–6746. 9822616 10.1093/emboj/17.22.6739 PMC1171019

[mpp12366-bib-0008] Chung, B.Y.‐W. , Miller, W.A. , Atkins, J.F. and Firth, A.E. (2008) An overlapping essential gene in the Potyviridae. Proc. Natl. Acad. Sci. USA, 105, 5897–5902. 18408156 10.1073/pnas.0800468105 PMC2311343

[mpp12366-bib-0009] Clark, C.A. , Davis, J.A. , Abad, J.A. , Cuellar, W.J. , Fuentes, S. , Kreuze, J.F. , Gibson, R.W. , Mukasa, S.B. , Tugume, A.K. and Tairo, F.D. (2012) Sweetpotato viruses: 15 years of progress on understanding and managing complex diseases. Plant Dis. 96, 168–185. 30731810 10.1094/PDIS-07-11-0550

[mpp12366-bib-0010] Cuellar, W.J. , Kreuze, J.F. , Rajamäki, M.‐L. , Cruzado, K.R. , Untiveros, M. and Valkonen, J.P. (2009) Elimination of antiviral defense by viral RNase III. Proc. Natl. Acad. Sci. USA, 106, 10 354–10 358. 19515815 10.1073/pnas.0806042106 PMC2694682

[mpp12366-bib-0011] El‐Shami, M. , Pontier, D. , Lahmy, S. , Braun, L. , Picart, C. , Vega, D. , Hakimi, M.‐A. , Jacobsen, S.E. , Cooke, R. and Lagrange, T. (2007) Reiterated WG/GW motifs form functionally and evolutionarily conserved ARGONAUTE‐binding platforms in RNAi‐related components. Genes Dev. 21, 2539–2544. 17938239 10.1101/gad.451207 PMC2000319

[mpp12366-bib-0012] Fang, Y. , Treffers, E.E. , Li, Y. , Tas, A. , Sun, Z. , van der Meer, Y. , de Ru, A.H. , van Veelen, P.A. , Atkins, J.F. and Snijder, E.J. (2012) Efficient −2 frameshifting by mammalian ribosomes to synthesize an additional arterivirus protein. Proc. Natl. Acad. Sci. USA, 109, E2920–E2928. 23043113 10.1073/pnas.1211145109 PMC3491471

[mpp12366-bib-0013] Firth, A.E. (2014) Mapping overlapping functional elements embedded within the protein‐coding regions of RNA viruses. Nucleic Acids Res. 42, 12 425–12 439. 10.1093/nar/gku981 PMC4227794 25326325

[mpp12366-bib-0014] Firth, A.E. and Brierley, I. (2012) Non‐canonical translation in RNA viruses. J. Gen. Virol. 93, 1385–1409. 22535777 10.1099/vir.0.042499-0 PMC3542737

[mpp12366-bib-0015] Gibson, R.W. , Aritua, V. , Byamukama, E. , Mpembe, I. and Kayongo, J. (2004) Control strategies for sweet potato virus disease in Africa. Virus Res. 100, 115–122. 15036842 10.1016/j.virusres.2003.12.023

[mpp12366-bib-0016] Giner, A. , Lakatos, L. , García‐Chapa, M. , López‐Moya, J.J. and Burgyán, J. (2010) Viral protein inhibits RISC activity by argonaute binding through conserved WG/GW motifs. PLoS Pathog. 6, e1000996. 20657820 10.1371/journal.ppat.1000996 PMC2904775

[mpp12366-bib-0017] Gitlin, L. , Hagai, T. , LaBarbera, A. , Solovey, M. and Andino, R. (2014) Rapid evolution of virus sequences in intrinsically disordered protein regions. PLoS Pathog. 10, e1004529. 25502394 10.1371/journal.ppat.1004529 PMC4263755

[mpp12366-bib-0018] Gutierrez, D. , Fuentes, S. and Salazar, L. (2003) Sweetpotato virus disease (SPVD): distribution, incidence, and effect on sweetpotato yield in Peru. Plant Dis. 87, 297–302. 30812764 10.1094/PDIS.2003.87.3.297

[mpp12366-bib-0019] Haikonen, T. , Rajamäki, M.‐L. and Valkonen, J.P. (2013) Interaction of the microtubule‐associated host protein HIP2 with viral helper component proteinase is important in infection with Potato virus A. Mol. Plant–Microbe Interact. 26, 734–744. 23489059 10.1094/MPMI-01-13-0023-R

[mpp12366-bib-0020] Haldeman‐Cahill, R. , Daròs, J.‐A. and Carrington, J.C. (1998) Secondary structures in the capsid protein coding sequence and 3′ nontranslated region involved in amplification of the tobacco etch virus genome. J. Virol. 72, 4072–4079. 9557696 10.1128/jvi.72.5.4072-4079.1998 PMC109636

[mpp12366-bib-0021] Hausmann, S. , Garcin, D. , Morel, A.‐S. and Kolakofsky, D. (1999) Two nucleotides immediately upstream of the essential A6G3 slippery sequence modulate the pattern of G insertions during Sendai virus mRNA editing. J. Virol. 73, 343–351. 9847338 10.1128/jvi.73.1.343-351.1999 PMC103839

[mpp12366-bib-0022] Hofacker, I.L. , Fekete, M. and Stadler, P.F. (2002) Secondary structure prediction for aligned RNA sequences. J. Mol. Biol. 319, 1059–1066. 12079347 10.1016/S0022-2836(02)00308-X

[mpp12366-bib-0023] Johansen, L.K. and Carrington, J.C. (2001) Silencing on the spot. Induction and suppression of RNA silencing in the Agrobacterium‐mediated transient expression system. Plant Physiol. 126, 930–938. 11457942 10.1104/pp.126.3.930 PMC1540124

[mpp12366-bib-0024] Kasschau, K.D. and Carrington, J.C. (1998) A counterdefensive strategy of plant viruses: suppression of posttranscriptional gene silencing. Cell, 95, 461–470. 9827799 10.1016/s0092-8674(00)81614-1

[mpp12366-bib-0025] Kelley, L.A. and Sternberg, M.J. (2009) Protein structure prediction on the Web: a case study using the Phyre server. Nat. Protoc. 4, 363–371. 19247286 10.1038/nprot.2009.2

[mpp12366-bib-0026] Kokkinos, C. and Clark, C. (2006) Interactions among Sweet potato chlorotic stunt virus and different potyviruses and potyvirus strains infecting sweetpotato in the United States. Plant Dis. 90, 1347–1352. 30780944 10.1094/PD-90-1347

[mpp12366-bib-0027] Kreuze, J.F. , Savenkov, E.I. , Cuellar, W. , Li, X. and Valkonen, J.P. (2005) Viral class 1 RNase III involved in suppression of RNA silencing. J. Virol. 79, 7227–7238. 15890961 10.1128/JVI.79.11.7227-7238.2005 PMC1112141

[mpp12366-bib-0028] Kreuze, J.F. , Perez, A. , Untiveros, M. , Quispe, D. , Fuentes, S. , Barker, I. and Simon, R. (2009) Complete viral genome sequence and discovery of novel viruses by deep sequencing of small RNAs: a generic method for diagnosis, discovery and sequencing of viruses. Virology, 388, 1–7. 19394993 10.1016/j.virol.2009.03.024

[mpp12366-bib-0029] Lakatos, L. , Szittya, G. , Silhavy, D. and Burgyán, J. (2004) Molecular mechanism of RNA silencing suppression mediated by p19 protein of tombusviruses. EMBO J. 23, 876–884. 14976549 10.1038/sj.emboj.7600096 PMC381004

[mpp12366-bib-0030] Larkin, M.A. , Blackshields, G. , Brown, N. , Chenna, R. , McGettigan, P.A. , McWilliam, H. , Valentin, F. , Wallace, I.M. , Wilm, A. and Lopez, R. (2007) Clustal W and Clustal X version 2.0. Bioinformatics, 23, 2947–2948. 17846036 10.1093/bioinformatics/btm404

[mpp12366-bib-0031] Li, F. , Xu, D. , Abad, J. and Li, R. (2012) Phylogenetic relationships of closely related potyviruses infecting sweet potato determined by genomic characterization of Sweet potato virus G and Sweet potato virus 2. Virus Genes, 45, 118–125. 22562225 10.1007/s11262-012-0749-2

[mpp12366-bib-0032] Li, H. and Durbin, R. (2009) Fast and accurate short read alignment with Burrows–Wheeler transform. Bioinformatics, 25, 1754–1760. 19451168 10.1093/bioinformatics/btp324 PMC2705234

[mpp12366-bib-0033] Lu, R. , Folimonov, A. , Shintaku, M. , Li, W.‐X. , Falk, B.W. , Dawson, W.O. and Ding, S.‐W. (2004) Three distinct suppressors of RNA silencing encoded by a 20‐kb viral RNA genome. Proc. Natl. Acad. Sci. USA, 101, 15 742–15 747. 10.1073/pnas.0404940101 PMC524217 15505219

[mpp12366-bib-0034] Mbanzibwa, D.R. , Tian, Y. , Mukasa, S.B. and Valkonen, J.P. (2009) Cassava brown streak virus (*Potyviridae*) encodes a putative Maf/HAM1 pyrophosphatase implicated in reduction of mutations and a P1 proteinase that suppresses RNA silencing but contains no HC‐Pro. J. Virol. 83, 6934–6940. 19386713 10.1128/JVI.00537-09 PMC2698530

[mpp12366-bib-0035] Olspert, A. , Chung, B.Y.W. , Atkins, J.F. , Carr, J.P. and Firth, A.E. (2015) Transcriptional slippage in the positive‐sense RNA virus family Potyviridae. EMBO Rep. 16, 995–1004. e201540509. 26113364 10.15252/embr.201540509 PMC4552492

[mpp12366-bib-0036] Pall, G.S. and Hamilton, A.J. (2008) Improved northern blot method for enhanced detection of small RNA. Nat. Protoc. 3, 1077–1084. 18536652 10.1038/nprot.2008.67

[mpp12366-bib-0037] Pardina, P.R. , Bejerman, N. , Luque, A.V. and Di Feo, L. (2012) Complete nucleotide sequence of an Argentinean isolate of sweet potato virus G. Virus Genes, 45, 593–595. 22826154 10.1007/s11262-012-0784-z

[mpp12366-bib-0038] Quevillon, E. , Silventoinen, V. , Pillai, S. , Harte, N. , Mulder, N. , Apweiler, R. and Lopez, R. (2005) InterProScan: protein domains identifier. Nucleic Acids Res. 33, W116–W120. 15980438 10.1093/nar/gki442 PMC1160203

[mpp12366-bib-0039] Rajamäki, M.‐L. , Kelloniemi, J. , Alminaite, A. , Kekarainen, T. , Rabenstein, F. and Valkonen, J.P. (2005) A novel insertion site inside the potyvirus P1 cistron allows expression of heterologous proteins and suggests some P1 functions. Virology, 342, 88–101. 16112702 10.1016/j.virol.2005.07.019

[mpp12366-bib-0040] Rancurel, C. , Khosravi, M. , Dunker, A.K. , Romero, P.R. and Karlin, D. (2009) Overlapping genes produce proteins with unusual sequence properties and offer insight into de novo protein creation. J. Virol. 83, 10 719–10 736. 10.1128/JVI.00595-09 PMC2753099 19640978

[mpp12366-bib-0041] Reeder, J. , Steffen, P. and Giegerich, R. (2007) pknotsRG: RNA pseudoknot folding including near‐optimal structures and sliding windows. Nucleic Acids Res. 35, W320–W324. 17478505 10.1093/nar/gkm258 PMC1933184

[mpp12366-bib-0067] Revers, F. and García, J.A. (2015) Chapter Three‐Molecular Biology of Potyviruses. Advances in virus research 92, 101–199. 25701887 10.1016/bs.aivir.2014.11.006

[mpp12366-bib-0042] Rice, P. , Longden, I. and Bleasby, A. (2000) EMBOSS: the European molecular biology open software suite. Trends Genet. 16, 276–277. 10827456 10.1016/s0168-9525(00)02024-2

[mpp12366-bib-0043] Rodamilans, B. , Valli, A. , Mingot, A. , San León, D. , Baulcombe, D. , López‐Moya, J.J. and García, J.A. (2015) RNA polymerase slippage as a mechanism for the production of frameshift gene products in plant viruses of the Potyviridae family. J. Virol. 89, 6965–6967. 10.1128/JVI.00337‐15. 25878117 10.1128/JVI.00337-15 PMC4468506

[mpp12366-bib-0044] Rohožková, J. and Navrátil, M. (2011) P1 peptidase—a mysterious protein of family Potyviridae. J. Biosci. 36, 189–200. 21451259 10.1007/s12038-011-9020-6

[mpp12366-bib-0045] de Ronde, D. , Pasquier, A. , Ying, S. , Butterbach, P. , Lohuis, D. and Kormelink, R. (2014) Analysis of *Tomato spotted wilt virus* NSs protein indicates the importance of the N‐terminal domain for avirulence and RNA silencing suppression. Mol. Plant Pathol. 15, 185–195. 24103150 10.1111/mpp.12082 PMC6638762

[mpp12366-bib-0046] Sabath, N. , Wagner, A. and Karlin, D. (2012) Evolution of viral proteins originated de novo by overprinting. Mol. Biol. Evol. 29, 3767–3780. 22821011 10.1093/molbev/mss179 PMC3494269

[mpp12366-bib-0047] Sakai, J. , Mori, M. , Morishita, T. , Tanaka, M. , Hanada, K. , Usugi, T. and Nishiguchi, M. (1997) Complete nucleotide sequence and genome organization of sweet potato feathery mottle virus (S strain) genomic RNA: the large coding region of the P1 gene. Arch. Virol. 142, 1553–1562. 9672618 10.1007/s007050050179

[mpp12366-bib-0048] Sambrook, J. and Russell, D.W. (2001) Molecular Cloning: A Laboratory Manual. Cold Spring Harbor, New York: Cold Spring Harbor Laboratory Press.

[mpp12366-bib-0049] Senshu, H. , Yamaji, Y. , Minato, N. , Shiraishi, T. , Maejima, K. , Hashimoto, M. , Miura, C. , Neriya, Y. and Namba, S. (2011) A dual strategy for the suppression of host antiviral silencing: two distinct suppressors for viral replication and viral movement encoded by potato virus M. J. Virol. 85, 10 269–10 278. 10.1128/JVI.05273-11 PMC3196401 21752911

[mpp12366-bib-0050] Shiboleth, Y.M. , Haronsky, E. , Leibman, D. , Arazi, T. , Wassenegger, M. , Whitham, S.A. , Gaba, V. and Gal‐On, A. (2007) The conserved FRNK box in HC‐Pro, a plant viral suppressor of gene silencing, is required for small RNA binding and mediates symptom development. J. Virol. 81, 13 135–13 148. 10.1128/JVI.01031-07 PMC2169133 17898058

[mpp12366-bib-0051] Söding, J. , Biegert, A. and Lupas, A.N. (2005) The HHpred interactive server for protein homology detection and structure prediction. Nucleic Acids Res. 33, W244–W248. 15980461 10.1093/nar/gki408 PMC1160169

[mpp12366-bib-0052] Szabó, E.Z. , Manczinger, M. , Göblös, A. , Kemény, L. and Lakatos, L. (2012) Switching on RNA silencing suppressor activity by restoring argonaute binding to a viral protein. J. Virol. 86, 8324–8327. 22623784 10.1128/JVI.00627-12 PMC3421681

[mpp12366-bib-0053] Tairo, F. , Mukasa, S.B. , Jones, R.A. , Kullaya, A. , Rubaihayo, P.R. and Valkonen, J. (2005) Unravelling the genetic diversity of the three main viruses involved in sweet potato virus disease (SPVD), and its practical implications. Mol. Plant Pathol. 6, 199–211. 20565651 10.1111/j.1364-3703.2005.00267.x

[mpp12366-bib-0054] Tatineni, S. , Qu, F. , Li, R. , Morris, T.J. and French, R. (2012) Triticum mosaic poacevirus enlists P1 rather than HC‐Pro to suppress RNA silencing‐mediated host defense. Virology, 433, 104–115. 22877841 10.1016/j.virol.2012.07.016

[mpp12366-bib-0055] Tena Fernández, F. , González, I. , Doblas, P. , Rodríguez, C. , Sahana, N. , Kaur, H. , Tenllado, F. , Praveen, S. and Canto, T. (2013) The influence of cis‐acting P1 protein and translational elements on the expression of *Potato virus Y* helper‐component proteinase (HCPro) in heterologous systems and its suppression of silencing activity. Mol. Plant Pathol. 14, 530–541. 23451733 10.1111/mpp.12025 PMC6638740

[mpp12366-bib-0056] Till, S. , Lejeune, E. , Thermann, R. , Bortfeld, M. , Hothorn, M. , Enderle, D. , Heinrich, C. , Hentze, M.W. and Ladurner, A.G. (2007) A conserved motif in Argonaute‐interacting proteins mediates functional interactions through the Argonaute PIWI domain. Nat. Struct. Mol. Biol. 14, 897–903. 17891150 10.1038/nsmb1302

[mpp12366-bib-0057] Tugume, A.K. , Mukasa, S.B. , Kalkkinen, N. and Valkonen, J.P. (2010) Recombination and selection pressure in the ipomovirus Sweet potato mild mottle virus (Potyviridae) in wild species and cultivated sweetpotato in the centre of evolution in East Africa. J. Gen. Virol. 91, 1092–1108. 19923261 10.1099/vir.0.016089-0

[mpp12366-bib-0058] Untiveros, M. , Fuentes, S. and Salazar, L.F. (2007) Synergistic interaction of Sweet potato chlorotic stunt virus (Crinivirus) with carla‐, cucumo‐, ipomo‐, and potyviruses infecting sweet potato. Plant Dis. 91, 669–676. 30780474 10.1094/PDIS-91-6-0669

[mpp12366-bib-0059] Untiveros, M. , Fuentes, S. and Kreuze, J. (2008) Molecular variability of sweet potato feathery mottle virus and other potyviruses infecting sweet potato in Peru. Arch. Virol. 153, 473–483. 18172571 10.1007/s00705-007-0019-0

[mpp12366-bib-0060] Untiveros, M. , Quispe, D. and Kreuze, J. (2010) Analysis of complete genomic sequences of isolates of the Sweet potato feathery mottle virus strains C and EA: molecular evidence for two distinct potyvirus species and two P1 protein domains. Arch. Virol. 155, 2059–2063. 20882307 10.1007/s00705-010-0805-y

[mpp12366-bib-0061] Valli, A. , Martín‐Hernández, A.M. , López‐Moya, J.J. and García, J.A. (2006) RNA silencing suppression by a second copy of the P1 serine protease of Cucumber vein yellowing ipomovirus, a member of the family Potyviridae that lacks the cysteine protease HCPro. J. Virol. 80, 10 055–10 063. 10.1128/JVI.00985-06 PMC1617295 17005683

[mpp12366-bib-0062] Valli, A. , López‐Moya, J.J. and García, J.A. (2007) Recombination and gene duplication in the evolutionary diversification of P1 proteins in the family Potyviridae. J. Gen. Virol. 88, 1016–1028. 17325376 10.1099/vir.0.82402-0

[mpp12366-bib-0063] Wang, M. , Abad, J. , Fuentes, S. and Li, R. (2013) Complete genome sequence of the original Taiwanese isolate of sweet potato latent virus and its relationship to other potyviruses infecting sweet potato. Arch. Virol. 158, 2189–2192. 23624656 10.1007/s00705-013-1705-8

[mpp12366-bib-0064] Xue, B. , Dunbrack, R.L. , Williams, R.W. , Dunker, A.K. and Uversky, V.N. (2010) PONDR‐FIT: a meta‐predictor of intrinsically disordered amino acids. Biochim. Biophys. Acta, 1804, 996–1010. 20100603 10.1016/j.bbapap.2010.01.011 PMC2882806

[mpp12366-bib-0065] Yamasaki, S. , Sakai, J. , Fuji, S. , Kamisoyama, S. , Emoto, K. , Ohshima, K. and Hanada, K. (2010) Comparisons among isolates of Sweet potato feathery mottle virus using complete genomic RNA sequences. Arch. Virol. 155, 795–800. 20336334 10.1007/s00705-010-0633-0

[mpp12366-bib-0066] Young, B.A. , Stenger, D.C. , Qu, F. , Morris, T.J. , Tatineni, S. and French, R. (2012) Tritimovirus P1 functions as a suppressor of RNA silencing and an enhancer of disease symptoms. Virus Res. 163, 672–677. 22230313 10.1016/j.virusres.2011.12.019

