# Supplementary figures and images for: A novel sweet potato potyvirus open reading frame (ORF) is expressed via polymerase slippage and suppresses RNA silencing
Source: Mol Plant Pathol. 2016 Apr 28;17(7):1111–23. doi: 10.1111/mpp.12366 (PMC4979677; doi:10.1111/mpp.12366)

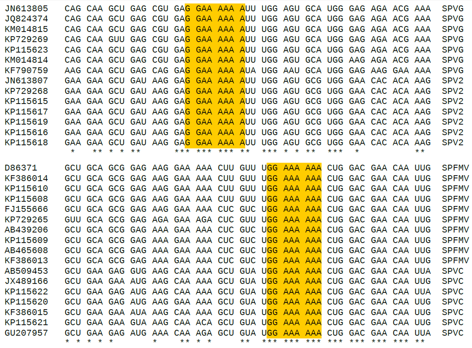

Supplement: Supplementary file 1 — Fig. S1 Predicted pispo G2A6 slippage sites. Predicted pispo G2A6 slippage sites (orange highlighted) in all available full‐length Sweet potato feathery mottle virus (SPFMV)‐group sequences. SPV2, Sweet potato virus 2; SPVC, Sweet potato virus C; SPVG, Sweet potato virus G. [file MPP-17-1111-s001.tif]

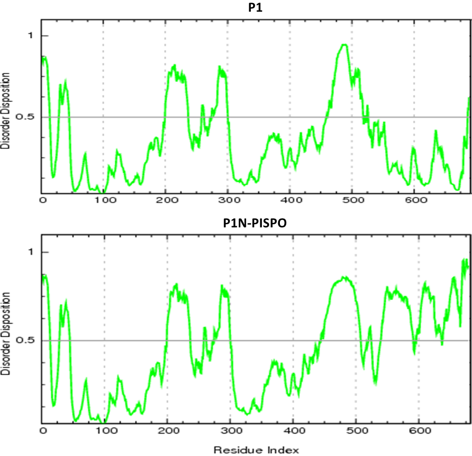

Supplement: Supplementary file 2 — Fig. S2 PONDR® disorder predictions for P1 and P1N‐PISPO. Common regions predicted to be unstructured or disordered include a small region of ∼25 residues within the N‐terminus of P1N, and the hypervariable region (residues 200–300). In P1, a third region of predicted disorder occurs around residues 459–522, whereas, in P1N‐PISPO, a third region of predicted disorder encompasses almost the entire PISPO domain starting around residue 444. Predicted non‐disordered domains have scores no higher than 0.5. [file MPP-17-1111-s002.tif]

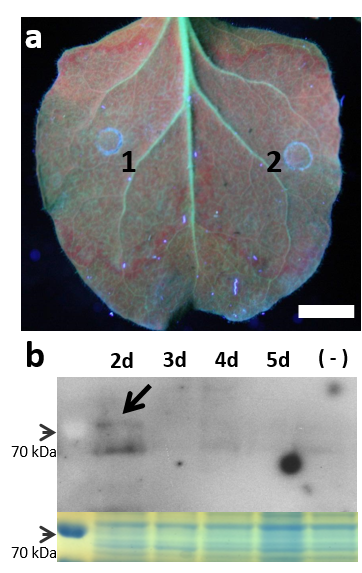

Supplement: Supplementary file 3 — Fig. S3 Expression and detection of YN‐P1(SPLV) in agroinfiltrated leaf tissue of green fluorescent protein (GFP)‐transgenic Nicotiana benthamiana line 16c. (a) Neither (1) YN‐P1(SPLV) (left half of the leaf) nor (2) YN‐HCpro (right half of the leaf) of Sweet potato feathery mottle virus (SPFMV) suppressed gfp silencing and no enhancement of GFP fluorescence was observed at 4 days post‐infiltration (dpi). The infiltrated tissue was surrounded by a red edge indicating short‐distance movement of the silencing signal. Scale bar indicates 2 cm. (b) Expressed YN‐P1(SPLV) protein was detected at 2 dpi using anti‐YN antibodies, but not at 3, 4 or 5 dpi. The upper band indicated with an arrow (2d) corresponds to the expected size (72 kDa) of YN‐P1(SPLV). The small arrow on the left indicates the position of the 70‐kDa protein marker. Staining of total proteins by Coomassie blue was used as a loading control. [file MPP-17-1111-s003.tif]
