# Supplementary material for: A novel sweet potato potyvirus open reading frame (ORF) is expressed via polymerase slippage and suppresses RNA silencing
Source: Mol Plant Pathol. 2016 Apr 28;17(7):1111–23. doi: 10.1111/mpp.12366 (PMC4979677; doi:10.1111/mpp.12366)
Supplement: Supplementary file 4 — Table S1 Mean amino acid inter‐ and intra‐species identities. Identities (%) calculated for the PISPO (italics; upper left) and P1‐pro (roman; lower right) domains. [file MPP-17-1111-s004.docx]

**Table S1.** **Mean amino acid inter- and intra-species identities.** Identities (%) calculated for the PISPO (*italics*; upper left) and P1-pro domains (roman; lower right).

|  | **SPFMV**  **O** | **SPFMV**  **Piu3** | **SPFMV**  **S** | **SPFMV**  **835** | **SPFMV**  **Ruk73** | **SPVC**  **Bongo** | **SPVC**  **C1** | **SPV2**  **LSU2** | **SPVG**  **LSU1** |
| --- | --- | --- | --- | --- | --- | --- | --- | --- | --- |
| **SPFMV O** | - | *91,4* | *71,2* | *69,8* | *92,3* | *41,4* | *37,4* | *28,8* | *30,6* |
| **SPFMV Piu3** | 93,9 | - | *69,8* | *69,4* | *89,6* | *42,3* | *38,7* | *31,1* | *29,3* |
| **SPFMV S** | 83,8 | 83,4 | - | *95,5* | *71,2* | *40,1* | *38,7* | *26,1* | *29,7* |
| **SPFMV 835** | 82,5 | 83,0 | 95,6 | - | *70,3* | *41,4* | *40,1* | *27,0* | *30,2* |
| **SPFMV Ruk73** | 95,2 | 93,9 | 82,1 | 81,7 | - | *40,1* | *37,8* | *29,3* | *31,5* |
| **SPVC Bongo** | 68,6 | 67,7 | 68,1 | 67,2 | 66,8 | - | *86,5* | *26,1* | *30,2* |
| **SPVC C1** | 68,6 | 67,7 | 66,8 | 65,9 | 68,1 | 93,4 | - | *24,3* | *27,9* |
| **SPV2 LSU2** | 53,3 | 52,4 | 50,7 | 49,8 | 53,3 | 48,0 | 47,2 | - | *28,8* |
| **SPVG LSU1** | 48,9 | 48,5 | 49,3 | 50,2 | 48,0 | 49,8 | 48,5 | 56,8 | - |
