# Supplementary material for: A novel sweet potato potyvirus open reading frame (ORF) is expressed via polymerase slippage and suppresses RNA silencing
Source: Mol Plant Pathol. 2016 Apr 28;17(7):1111–23. doi: 10.1111/mpp.12366 (PMC4979677; doi:10.1111/mpp.12366)
Supplement: Supplementary file 5 — Table S2 Virus species and accession number for sequences used for the computational analysis of the pispo open reading frame (ORF). Sequences of Sweet potato virus G (SPVG)‐LSU1 and Sweet potato virus 2 (SPV2)‐LSU2 were determined in this study, and partial sequences with coverage of pispo also included are indicated in a separate column. [file MPP-17-1111-s005.docx]

S2 Table. Virus species and accession number for sequences used for the computational analysis of the *pispo* ORF. Sequences of SPFMV-Ruk73, SPVG-LSU1 and SPV2- LSU2 were determined in this study, and partial sequences with coverage of pispo also included are indicated in a separate column.

| SPFMV | SPVC | SPVG | SPV2 | Partial |
| --- | --- | --- | --- | --- |
| KP729265.1 (Ruk73), AB439206.1,  AB465608.1,  FJ155666.1,  D86371.1,  KP115608.1,  KP115609.1,  KP115610.1,  KF386013.1,  KF386014.1; | AB509453.1, , JX489166.1,  GU207957.1,  KF386015.1,  KP115620.1,  KP115621.1,  KP115622.1 | KP729269.1 (LSU1), JN613805.1,  JN613806.1 (identical to JN613805.1),  JQ824374.1,  KF790759.1,  KM014814.1,  KM014815.1,  KP115623.1 | KP729268.1 (LSU2), JN613807.1,  KP115615.1,  KP115616.1,  KP115617.1,  KP115618.1,  KP115619.1 | AB509455.1, AB509457.1,  AB509459.1,  AB509461.1,  AB509463.1,  AY523538.1,  AY523541.1,  AY523546.1,  GU207949.1,  GU207950.1, GU207951.1, GU207954.1, GU207955.1, JQ742091.1 |
