# Supplementary material for: A novel sweet potato potyvirus open reading frame (ORF) is expressed via polymerase slippage and suppresses RNA silencing
Source: Mol Plant Pathol. 2016 Apr 28;17(7):1111–23. doi: 10.1111/mpp.12366 (PMC4979677; doi:10.1111/mpp.12366)
Supplement: Supplementary file 6 — Table S3 Primer sequences used in this study. [file MPP-17-1111-s006.docx]

**Table S3. Oligonucleotides used in the present study.** Oligonucleotides were designed based on SPFMV isolate Ruk73 (KP729265.1), and SPLV isolate Taiwan (KC443039.1) sequences. Nucleotide substitutions are underlined, insertions are in bold, and restriction enzymes sites are in italics. Oligonucleotides were obtained from Oligomer (Helsinki, Finland).

| **Name** | **Sequence (5’-3’)** | **Target** |
| --- | --- | --- |
| P1 Start I | aatt*tctaga*atggcaaccgttattgcatctgt | P1 SPFMV cloning |
| P1 End I | ttaa*cccggg*ctaaaactgagtgatatatggcaggac | P1 SPFMV cloning |
| P1N + pispo F | gagagagaaagactcgtttggaagaag**a**ctggacgaacaattggctgc | Frameshift for PISPO expression |
| P1N + pispo R | gcagccaattgttcgtccag**t**cttcttccaaacgagtctttctctctc | Frameshiftfor PISPO expression |
| P1∆pispo F | ggacgaacaattggctgctaggaacgaggccagaaaggacct | PISPO deletion |
| P1∆pispo R | aggtcctttctggcctcgttcctagcagccaattgttcgtcc | PISPO deletion |
| P1 ∆WG (1) F | gaatgctgcaacaaggcggggaaagctgcaatggaa | P1N WG #1 motif deletion |
| P1 ∆WG (1) R | ttccattgcagctttccccgccttgttgcagcattc | P1N WG #1 motif deletion |
| PISPO ∆WG (2) F | agtatctccatcgcgcggggtcctgcagcgag | PISPO WG #2 motif deletion |
| PISPO ∆WG (2) R | ctcgctgcaggaccccgcgcgatggagatact | PISPO WG #2 motif deletion |
| PISPO ∆WG (3) F | gcaagccgccaaggtgcgggcatgggagaagtcaat | PISPO WG #3 motif deletion |
| PISPO ∆WG (3) R | attgcattctcccatgcccgcaccttggcggcttgc | PISPO WG #3 motif deletion |
| PISPO ∆WG (4) F | aatgttcgtggtacggcgggctttcaaaggagtgtt | PISPO WG #4 motif deletion |
| PISPO ∆WG (4) R | aacactcctttgaaagcccgccgtaccacgaacaat | PISPO WG #4 motif deletion |
| HC-Pro SPFMV F | ttaa*tctaga*atgagttccaccttggaaaggtttctgtcagg | HC-pro SPFMV cloning |
| HC-Pro SPFMV R | aattcccgggtcaaccaactatatagtgcttcatttcagattccaaggaactac | HC-pro SPFMV cloning |
| P1 SPLV F | ttaa*tctaga*atggcagctgtaatttttggat | P1 SPLV cloning |
| P1 SPLV R | aatt*cccggg*tcagtaatgttgcatacggagcatcacagattcggtcactctc | P1 SPLV cloning |
| pRTYN F | gacc*tctaga*atggtgagcaagggcgag | YN tagging |
| pRTYN R | ttaa*tctaga*ccatgccatgatatagacgttgtg | YN tagging |
